# Epidemiology and Genetic Characterization of Distinct Ebola Sudan Outbreaks in Uganda

**DOI:** 10.3390/idr17030044

**Published:** 2025-05-01

**Authors:** Francesco Branda, Massimo Ciccozzi, Fabio Scarpa

**Affiliations:** 1 Unit of Medical Statistics and Molecular Epidemiology, Università Campus Bio-Medico di Roma, 00128 Rome, Italy; m.ciccozzi@unicampus.it; 2 Department of Biomedical Sciences, University of Sassari, 07100 Sassari, Italy; fscarpa@uniss.it

**Keywords:** Ebola Sudan virus (SUDV), Sudan virus (SUDV), Uganda outbreaks, epidemiology, genetic characterization, phylogenomic analysis, transmission dynamics, viral evolution, public health response

## Abstract

**Background**. Sudan virus (SUDV) has caused multiple outbreaks in Uganda over the past two decades, leading to significant morbidity and mortality. The recent outbreaks in 2022 and 2025 highlight the ongoing threat posed by SUDV and the challenges in its containment. This study aims to characterize the epidemiological patterns and phylogenomic evolution of SUDV outbreaks in Uganda, identifying key factors influencing transmission and disease severity. **Methods**. We conducted a retrospective observational study analyzing epidemiological and genomic data from SUDV outbreaks in Uganda between 2000 and 2025. Epidemiological data were collected from official sources, including the Ugandan Ministry of Health and the World Health Organization, supplemented with reports from public health organizations. Genomic sequences of SUDV were analyzed to investigate viral evolution and identify genetic variations associated with pathogenicity and transmissibility. **Results**. The 2022 outbreak involved 164 confirmed cases and a case fatality rate (CFR) of 33.5%, with significant geographic variation in case distribution. The 2025 outbreak, still ongoing, was first detected in Kampala, with evidence of both nosocomial and community transmission. Phylogenomic analysis revealed the presence of two main genetic groups, representing Sudan and Uganda, respectively. The genetic variability of the Ugandan cluster is higher than that observed in Sudan, suggesting a greater expansion potential, which aligns with the current outbreak. Epidemiological findings indicate that human mobility, weaknesses in the health system, and delays in detection contribute to the amplification of the outbreak. **Conclusions**. Our findings underscore the importance of integrated genomic and epidemiological surveillance in understanding SUDV transmission dynamics. The recurrent emergence of SUDV highlights the need for improved outbreak preparedness, rapid response mechanisms, and international collaboration. Strengthening real-time surveillance and enhancing healthcare system resilience are critical to mitigating the impact of future outbreaks.

## 1. Introduction

Sudan virus (SUDV), formerly known as Ebola Sudan Virus [1], is a members of the genus *Orthoebolavirus* and is responsible for recurrent epidemic outbreaks in sub-Saharan Africa, characterized by high lethality and significant socioeconomic impact [2]. SUDV is genetically distinct from other members of the Orthoebolavirus genus, with a nucleotide divergence of about 35–45% compared to the well-known *Orthoebolavirus zairense* [3]. More importantly, it also shows differences in antigenicity and pathogenicity [4]. This places it in a separate clade in the *Orthoebolavirus* phylogenetic tree. Phylogenetic analysis of outbreaks has revealed that SUDV evolves at a mutation rate similar to other species of *Orthoebolavirus*, estimated to be around 8 × 10^−4^ substitutions per site per year [5]. However, unlike EBOV, which showed a greater capacity for adaptation and human-to-human transmission during the 2014–2016 outbreak, SUDV has historically caused more localized outbreaks with lower transmissibility [6]. Although further investigation is needed to fully understand the transmission dynamics, the genetic variability observed among various SUDV strains from different outbreaks suggests that these events may have emerged independently, rather than indicating sustained human-to-human transmission, though this hypothesis requires additional data and confirmation. This supports the hypothesis of a natural reservoir for the virus, likely in fruit bats, although the exact reservoir has not yet been confirmed [7]. Historically, SUDV outbreaks have been less frequent than EBOV outbreaks but have still caused major epidemics, such as those in Uganda in 2000, 2011, and 2014 [8]. Unlike EBOV, which saw widespread during the 2014–2016 West African outbreak [9], SUDV has remained predominantly confined to specific regions, with limited but highly lethal transmission. In 2022, Uganda experienced a new outbreak of SUDV, characterized by rapid spread in several regions of the country and a mortality rate of more than 34% [10]. This outbreak posed a significant challenge to local health systems, highlighting the need for a rapid and coordinated response. Three years later, in 2025, a second SUDV outbreak emerged in Uganda, raising questions about transmission dynamics, viral evolution, and outbreak response capabilities [11]. The aim of our research is to provide an integrative perspective that considers both epidemiological and genetic data in order to enhance our understanding of the current outbreak.

## 2. Materials and Methods

### 2.1. Study Design and Population

We conducted a retrospective observational study to analyze the epidemiological trends, transmission dynamics, and genomic characteristics of SUDV outbreaks in Uganda from 2000 to 2025. The study population includes all laboratory-confirmed SUDV cases reported in this time frame, with detailed stratification by geographic location and occupational exposure (e.g., healthcare workers). Our analysis focuses on spatiotemporal variations in outbreak severity and case fatality rates (CFRs) by district, exploring patterns over time and space to identify high-risk areas. Moreover, to explore the mechanisms driving outbreak persistence and viral adaptation, we supplement epidemiological surveillance with high-resolution phylogenomic analyses. This allows us to perform a comparative assessment of viral evolution over multiple epidemic periods, identifying potential genetic mutations associated with changes in transmissibility, pathogenicity, or immune escape.

### 2.2. Sampling and Data Collection

Data collection follows standardized epidemiological and genomic protocols to ensure consistency across different outbreak periods. Retrospective data sources include a range of different sources to update and curate our open-access database [12]. First, for the epidemiological data, we use official government sources that report primary data as the gold standard for data inclusion. Government sources include press releases on the official websites of Ministries of Health and World Health Organization African Region, as well as updates provided by the official social media accounts of governmental or public health institutions. Second, to find additional details for each case or patient we augment these data with online reports, mainly captured through the Center for Infectious Disease Research and Policy (CIDRAP), a leading global resource for infectious disease news and analysis (https://www.cidrap.umn.edu/ebola, accessed on 10 February 2025) or via news aggregators (https://bnonews.com/index.php/tag/ebola, accessed on 10 February 2025).

Genomic sequences of SUDV were analyzed to investigate viral evolution, with comparisons to previous outbreak strains. The genomic dataset was built by downloading all available sequences from the Pathoplexus databank, section “Ebola Sudan” (https://pathoplexus.org/ebola-sudan/search?, accessed on 10 February 2025). The initial dataset consisted of 165 genomic sequences isolated between 1976 and 19 January 2025. The entire dataset was aligned using the MAFFT (v7.505) [13] software and manually checked with UGene Pro (v35) [14]. After the alignment, the dataset was further filtered to remove short and uninformative sequences. The final dataset, with a length of 18,875 bp, consisted of 142 highly informative sequences. To explore the variability of SUDV lineages over an extended timeframe, all genomes were analyzed using Bayesian Inference (BI) with BEAST (v1.10.4) [15]. The analysis involved 200 million generations under different demographic and clock models. The most suitable model was selected based on Bayes Factor testing [16], comparing 2lnBF values of marginal likelihoods using the software Tracer (v1.7) [17]. The obtained tree was edited and visualized using FigTree (v1.4.0) (http://tree.bio.ed.ac.uk/software/figtree/, accessed on 10 February 2025). In order to examine genetic structure, detect potential subgroups within genetic clusters, and evaluate genetic variability among isolates, the Principal Coordinate Analysis (PCoA) was conducted using GenAlEx (v6.5) [18]. This analysis aimed to measure dissimilarity based on the genetic variation present in the analyzed isolates. The PCoA reconstruction relied on a pairwise p-distance matrix derived from genetic data, estimated over 1000 iterations using the R package APE [19]. The analysis was finalized by applying the PCoA method through a covariance matrix with data standardization.

## 3. Results

### 3.1. Epidemiological Analysis

Uganda experienced two major outbreaks of Sudan virus disease (SVD) within three years, one in 2022–2023 and the other more recent in 2025. Although both epidemics were caused by the same viral species, they differed significantly in size, duration, geographic distribution, and epidemiological dynamics, as summarized in Table 1.

The 2025 outbreak, which was officially declared on 30 January 2025 [11], was characterized by a notably shorter duration than the 2022–2023 outbreak. The latter, which began on 20 September 2022, was declared over on 11 January 2023, after a 42-day period of no new confirmed cases, culminating in a total duration of approximately 114 days [20]. In contrast, the 2025 outbreak was initially declared contained by 18 February 2025, only to be followed by the emergence of new cases by 24 February 2025, thus suggesting a more complex transmission dynamic and the difficulty in effectively interrupting chains of transmission despite early containment efforts. This re-emergence of cases in 2025 illustrates the need for a more cautious approach when declaring an outbreak under control, particularly when complete interruption of transmission chains has not been verified through robust surveillance [21].

**Figure 1:**
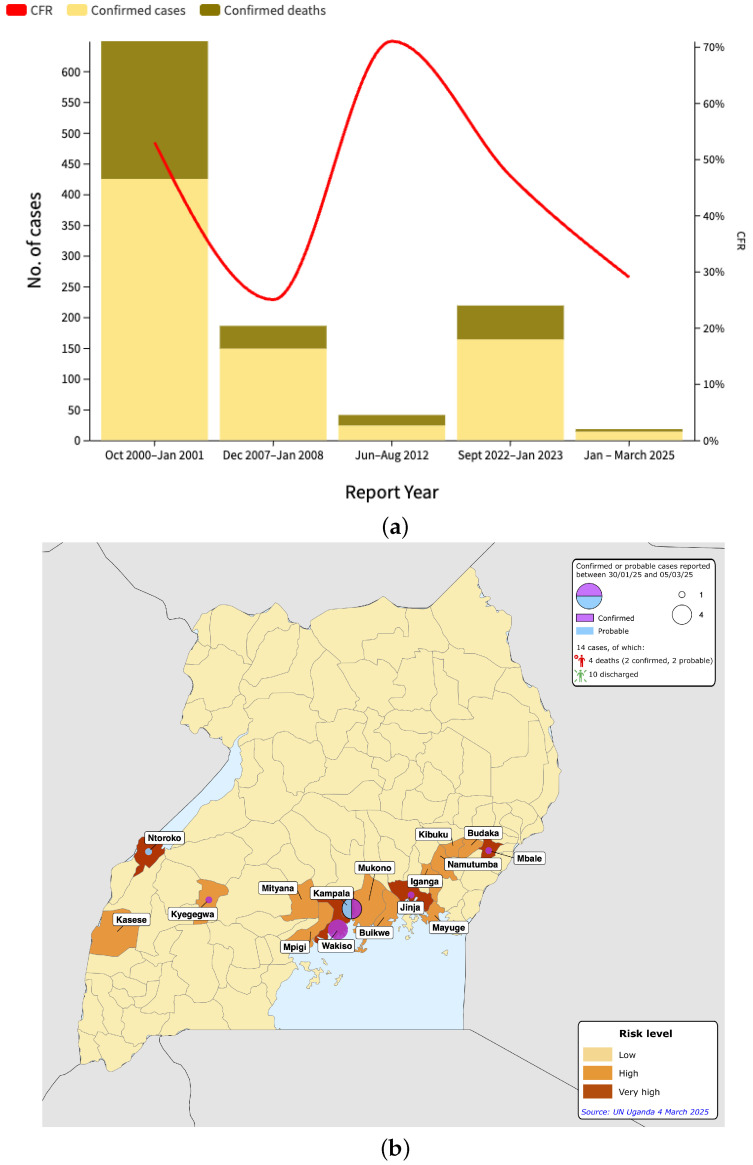

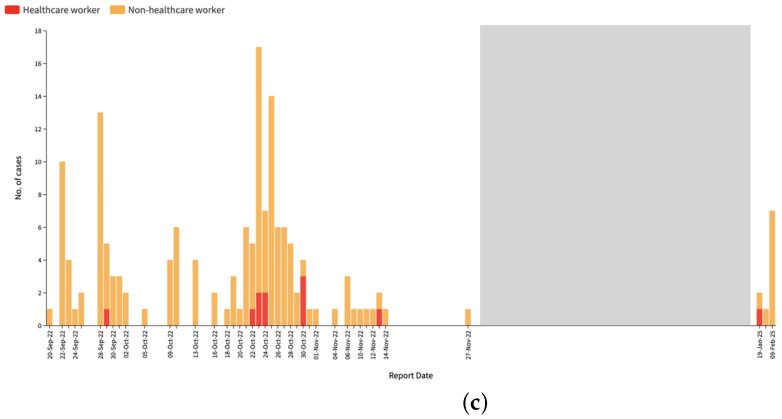
(**a**) Historical outbreaks of Ebola have occurred in Uganda. (**b**) Geographical distribution of confirmed Ebola cases and deaths since the last outbreak in 2025 by districts in Uganda. Adapted from “Uganda|Sudan (Ebola) Virus Disease outbreak and EU response” figure in [26]. (**c**) Number of confirmed cases of SUDV disease among healthcare workers and non-healthcare workers. The grey shading indicates periods from 2022 to 18 January 2025 when no cases were reported.

In terms of the number of confirmed cases, the 2025 epidemic reported a total of 14 cases, including 12 confirmed and 2 probable, as of 5 March 2025 [22]. This data represents a much smaller size of the epidemic than the 2022–2023 epidemic, which reported 164 cases, of which 142 were confirmed and 22 were probable [12]. The difference in the number of cases between the two outbreaks can be attributed to several factors, including geographic context, the timeliness and effectiveness of early containment efforts, and possible variations in virus transmissibility. In the 2025 outbreak, the initial cluster of cases appeared relatively small compared to the 2022–2023 outbreak, which expanded rapidly, particularly in the rural Mubende district, the epicenter of the outbreak [12]. Despite the reduced number of cases in 2025, the CFR remained alarmingly high (about 29%), with 4 deaths recorded as of 5 March 2025 [22] (Figure 1a). Although this value is lower than the CFR of 47% observed in the 2022–2023 outbreak [12], which resulted in more severe morbidity and mortality, it still highlights the high lethality associated with Sudan virus disease, a strain of Ebola virus with a historically high mortality rate [10]. The death of a four-year-old child in 2025 [23], underscores the vulnerability of children to severe outcomes of the disease, as was also observed in the 2022–2023 outbreak, in which 23 cases of Ebola were confirmed among school students, contributing to school closures nationwide.

The geographic distribution of cases in the 2025 outbreak shows a more limited spread than in the 2022–2023 outbreak. As of 5 March 2025, confirmed and probable cases in the 2025 outbreak were concentrated in six districts-Kampala, Wakiso, Mbale, Jinja, Mukono, and Ntoroko (Figure 1b) [22]. A notable feature of the 2025 outbreak was the identification of a second cluster in Ntoroko district, geographically distinct and with no known epidemiological link to the initial cases in Wakiso and Kampala. This second cluster raised concerns about undetected transmission chains operating in different regions of Uganda, highlighting the challenges of monitoring and controlling the spread of the virus beyond the initial epicenter. The 2022–2023 outbreak, on the other hand, affected nine districts [12], including Mubende, Kampala, and Wakiso, and required the imposition of severe movement restrictions, including closure in highly affected areas such as Mubende and Kassanda. The geographic spread of the 2022–2023 outbreak, which affected both rural areas and urban centers such as Kampala [24], was further exacerbated by the implementation of blocking and other containment measures. These measures, aimed at containing transmission, highlight the wide community spread observed during the outbreak, which likely contributed to its prolonged duration and the challenges faced in controlling its spread.

The demographic characteristics of the two outbreaks provide additional insight into the evolving epidemiology of SVD in Uganda. In the 2025 outbreak, the confirmed cases spanned a broad age range from 1.5 to 55 years, with a mean age of 27 years, and males accounted for 55% of cases [22]. This age distribution was somewhat consistent with the 2022–2023 outbreak, where young adults aged 20–29 years represented the largest proportion of cases (28%), followed closely by those aged 30–39 years (26%) [20]. In addition, males accounted for 58% of the cases, reflecting a similar sex distribution to that observed in the 2025 outbreak [20]. Healthcare workers were also significantly affected in both epidemics, with a substantial percentage of confirmed cases in 2025 (50%), and at least 19 healthcare workers infected, with seven deaths in the 2022–2023 epidemic (Figure 1c). Despite the lethal nature of the virus, infection can be prevented through the use of appropriate personal protective equipment (PPE) [25], which provides a key barrier against infectious body fluids, especially during direct contact with patients. However, the effectiveness of PPE is greatly enhanced when combined with other control measures, such as proper maintenance of healthcare facilities, safe waste management, and strict adoption of infection prevention and control protocols.

### 3.2. Phylogenomic Analysis

**Figure 2:**
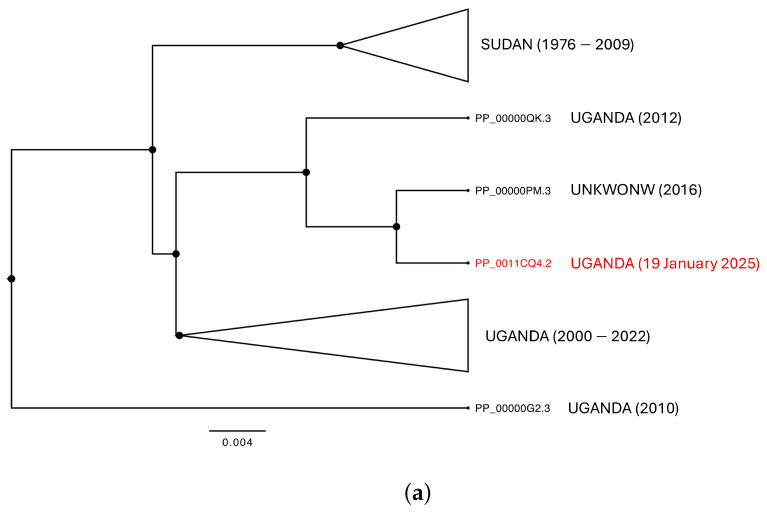

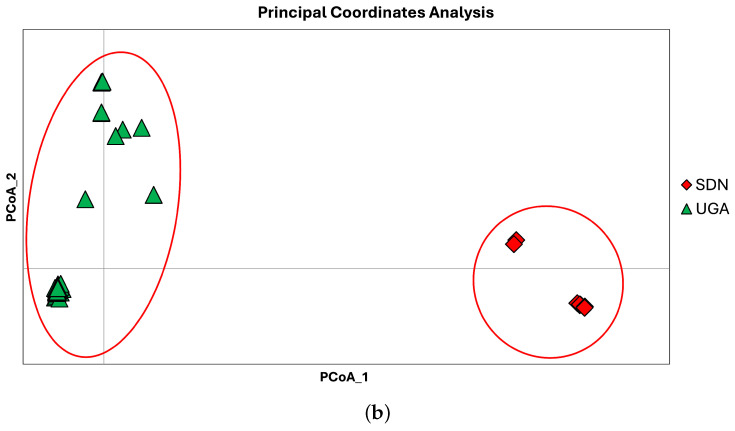
(**a**) Phylogenetic tree of all genomes of SUDV. All nodes are fully supported for Posterior Probabilities (PP). The terminal labeled in red font represent the only one available for the new current outbreak in Uganda. (**b**) Principal coordinate analysis of all genomes of SUDV. The cumulative variability explained by the first three axes amounts to 92.97% (PCoA_1: 43.85%, PCoA_2: 21.22%, PCoA_3: 18.89%). The groups were set a priori in accordance with the sampling locality.

Phylogenomic analyses identify two main genetic clusters representing the genetic variability of Sudan and Uganda, respectively (Figure 2a). These clusters reflect genetic patterns rather than being exclusive to a specific geographic area. The Sudanese cluster includes samples collected between 1976 and 2009. In contrast, the Ugandan cluster is divided into two groups: one more recent, composed of isolates collected between 2000 and 2022, and another consisting of three strains isolated in 2012, 2016, and one from the current outbreak, collected on 19 January 2025. The geographical structure and the genetic differentiation shaping the two main clusters has been confirmed by PCoA analyses, which explain a cumulative variability of 92.97% across the three main axes (Axis 1: 43.85%, Axis 2: 21.22%, Axis 3: 18.89%) (Figure 2b). The genetic differentiation between the two main clusters amounts to 0.055, between the two Ugandan groups to 0.010, and within the largest Ugandan group to 0.07. The evolutionary rate, estimated using a subset of samples with complete collection dates, is 3 × 10^−5^ (95% CI: 1.2308 × 10^−5^–4.3042 × 10^−5^).

## 4. Discussion

The epidemiological characterization of the SUDV outbreaks in Uganda highlights significant variations in transmission dynamics, case fatality rates, and geographic spread over the past two decades. Through our research team’s commitment to building and maintaining a comprehensive open-access database, we have been able to track and analyze outbreak data in real-time, providing crucial insights into the evolution of these epidemics.

Our analysis of previous outbreaks, particularly those in 2022, reveals a recurrent pattern of localized transmission with occasional spillover into urban centers, necessitating robust surveillance and early response mechanisms. The latest outbreak in 2025, with an index case identified in Kampala, underscores the persistent risk of viral re-emergence and the challenges in controlling transmission in densely populated areas. CFRs observed in different outbreaks have varied substantially, from 52.7% in the 2000 outbreak to 33.5% in the 2022 outbreak. These changes can be attributed to improvements in clinical management, more effective detection and reporting mechanisms, and increased public health preparedness. Although containment measures have led to significant progress, the detection of cases across multiple districts—including among healthcare workers—highlights the ongoing complexity of managing SUDV transmission, particularly in urban settings. These findings suggest that nosocomial transmission may still be contributing to the spread, underscoring the need for continued vigilance and assessment of infection control protocols. The role of human mobility in epidemic dynamics is particularly evident in the 2025 epidemic. The index case was a 32-year-old nurse, a resident of Wakiso district, who developed symptoms on 19 January 2025, and died on January 29 in Kampala. During the symptomatic period, he sought care from a traditional healer in Mbale district and visited three different health facilities: one in Wakiso district, one in Mbale district, and one in Kampala. The source of the Sudan virus exposure is still under investigation [27]. This underscores the need for improved case identification and contact tracing, particularly among frontline workers who are at greater risk of exposure. The rapid identification of more than 230 contacts, including symptomatic individuals who required isolation, suggests that Uganda’s public health system has made progress in responding to the outbreak. However, the delays in case detection observed in the early phase of this outbreak highlight the need to strengthen real-time surveillance and diagnostic capabilities.

Comparing the epidemiological profiles of these outbreaks, it is clear that although SUDV has remained largely confined to specific geographic regions, the risk of wider spread remains high. Urbanization, increased connectivity between districts and health care-seeking behaviors contribute to the complexity of containing outbreaks. In addition, the involvement of traditional healers, as seen in the 2025 outbreak, presents an additional challenge to ensure timely medical intervention and prevent further spread.

Another key finding of this study is the importance of integrating genomic surveillance with epidemiological surveys. While epidemiological data provide insights into transmission dynamics, genomic analyses are critical to understanding viral evolution and potential changes in virulence or transmissibility. The combination of these approaches will improve the ability to predict and mitigate future outbreaks. The phylogenomic analyses conducted in this study reveal a clear genetic structure within the SUDV lineage, distinguishing two main clusters representing samples from Sudan and Uganda. These clusters are indicative of genetic divergence but are not strictly confined to specific geographic regions, suggesting ongoing viral evolution and potential interregional transmission events. The Sudanese cluster encompasses isolates spanning over three decades (1976–2009). In contrast, the Ugandan cluster displays a more complex structure, divided into two subgroups: one comprising more recent isolates (2000–2022) and another containing three strains from 2012, 2016, and the most recent sample from the current outbreak (19 January 2025). This split suggests distinct transmission chains or evolutionary trajectories within Uganda. While the observed divergence could in part reflect viral diversity in geographically separated non-human reservoirs, the temporal and spatial clustering of certain lineages also points to a potential contribution from localized outbreaks and transmission dynamics in the human population. The genetic differentiation between the Sudanese and Ugandan clusters is quantified at 0.055, suggesting a moderate degree of divergence, potentially resulting from geographical separation and independent evolutionary pressures. Within Uganda, the differentiation between the two subgroups is lower (0.010), implying a more recent common ancestry or ongoing genetic exchange. Notably, the largest Ugandan group exhibits an internal differentiation of 0.07, indicating a significant level of genetic diversity, possibly due to recurrent introductions or mutations accumulating over time. The observed differences between the Sudanese and Ugandan lineages suggest distinct evolutionary trajectories. While outbreaks typically resolve swiftly, accumulating mutations during human-to-human transmission have been documented, indicating that transmission dynamics have influenced viral diversification. Additionally, the presence of similar variants in previous outbreaks points to a common zoonotic origin, highlighting the role of animal reservoirs in the observed genetic diversity. The higher differentiation within the largest Ugandan group may indicate the presence of multiple circulating lineages within the country, which could have implications for viral transmission and immune escape. Such genetic distances align with previous studies on filoviruses, where geographic and host factors contribute to evolutionary dynamics [28]. Although the Ugandan subgroups appear to show relatively lower genetic differentiation, this may reflect recent localized outbreaks or transmission bottlenecks. However, the apparent divergence patterns should be interpreted cautiously, given the limited genomic data available for the 1976 Sudan outbreak. The estimated evolutionary rate of Sudan virus (i.e., 3 × 10^−5^ substitutions per site per year) is consistent with previous estimates for filoviruses and suggests a relatively stable molecular clock. However, the observed genetic differentiation and clustering patterns highlight the potential for viral adaptation and evolution, which could impact outbreak dynamics, transmission efficiency, and vaccine or therapeutic effectiveness. As a consequence, the spread of the virus that is not uniform, with some areas serving as epicenters of infection and others recording only sporadic but still significant cases to understand the mechanisms of propagation. A central issue is the link between the mobility of individuals and the ability of the virus to spread beyond initially affected areas. Travel for health or personal reasons is a crucial vector in transmission, highlighting the need for targeted strategies for contact tracing and early containment. In addition, the recurrence of outbreaks raises questions about the persistence of the virus in the environment or potential animal reservoirs, suggesting the need for an integrated approach that combines human and veterinary surveillance. In contrast to the occurrence of Ebola outbreaks in other parts of Africa, there is no evidence of continuous transmission of Sudan virus (SUDV) between the 1976 and 2004 outbreaks. The reasons for this apparent interruption in viral circulation over a span of 28 years could be linked to ecological factors, such as the possible temporary disappearance of the natural reservoir, presumably fruit bats, which may have ceased to host the virus for indefinite periods. Additionally, the absence of documented outbreaks could also be attributed to the difficulty of detecting Ebola epidemics in remote areas where health surveillance may be limited. Another important factor to consider is that SUDV outbreaks, being spillover events, could occur under specific ecological conditions that may not have been repeated between the two events. Indeed, while the virus was detected during the 2004 outbreak, there is insufficient evidence to suggest continuous transmission from 1976 to 2004. Therefore, further ecological and virological studies, including animal surveillance and research on viral reservoirs, are necessary to better understand the factors influencing the emergence of new outbreaks and the modes of SUDV transmission. Although response measures have improved over time, critical issues persist related to delays in initial diagnosis and management of infections in hospital settings, factors that can amplify nosocomial transmission and complicate outbreak control. Beyond its impact in Africa, we emphasize that Ebola is not solely a regional concern but a potential global threat. With increasing international travel and human mobility, the risk of spillover events beyond endemic regions has grown, underscoring the need for robust global surveillance, rapid response mechanisms, and coordinated international efforts to prevent future outbreaks.

## 5. Conclusions

These findings highlight the descriptive value of genomic surveillance in tracking the evolutionary dynamics of Sudan virus. While the identification of distinct genetic clusters and geographic outliers provides important epidemiological insight, the current data do not allow us to infer functional consequences of the observed genetic variation. Further studies are needed to assess whether these variations are associated with phenotypic changes or simply represent neutral genetic drift. Future studies should focus on whole-genome sequencing of newly emerging strains, integration of epidemiological data, and assessment of phenotypic changes associated with genetic mutations to better understand the implications of viral evolution on public health interventions. Furthermore, sustained investment in epidemic preparedness is crucial, including community involvement, training of healthcare workers, and expansion of rapid diagnostic capabilities. Future outbreak response strategies must prioritize early detection, cross-border coordination, and the development of targeted interventions to mitigate the impact of SUDV in Uganda and elsewhere. The recurrent nature of SUDV outbreaks suggests that continuous surveillance and adaptive public health measures are essential to prevent large-scale epidemics and minimize the burden of this deadly virus.

## Figures and Tables

**Table 1:** Comparison between the 2025 and 2022–2023 SUDV outbreaks.

Indicator	2025 Outbreak (as of 5 March 2025)	2022–2023 Outbreak
Confirmed Cases	12	142
Probable Cases	2	22
Total Cases	14	164
Confirmed Deaths	2	55
Probable Deaths	2	22
Total Deaths	4	77
CFR	29%	47% (overall), 39% (confirmed)
Affected Districts	Jinja, Kampala, Kyegegwe, Mbale, Ntoroko, Wakiso	Bunyangabu, Jinja, Kagadi, Kampala, Kassanda, Kyegegwa, Masaka, Mubende, Wakiso
Duration	30 January 2025–ongoing	20 September 2022–11 January 2023
Mean Age of Cases	27 years	20–29 years (most affected)
Gender Distribution	55% Male	59% Male

## Data Availability

The data presented in this study are available at https://github.com/fbranda/ebola (accessed on 8 March 2025). Epidemiological data were extracted from publicly available reports published in the World Health Organization (WHO) Disease Outbreak News, using the keywords “Sudan virus disease—Uganda” (accessed on 8 March 2025). Genomic data were obtained from https://pathoplexus.org/ebola-sudan/search (accessed on 10 February 2025).
